# Factors Explaining Language Performance After Training in Elders With and Without Subjective Cognitive Decline

**DOI:** 10.3389/fnagi.2018.00264

**Published:** 2018-09-03

**Authors:** Ramón López-Higes, Jose M. Prados, Susana Rubio-Valdehita, Inmaculada Rodríguez-Rojo, Jaisalmer de Frutos-Lucas, Mercedes Montenegro, Pedro Montejo, David Prada, María L. D. Losada

**Affiliations:** ^1^Department of Experimental Psychology, Complutense University of Madrid, Madrid, Spain; ^2^Laboratory of Cognitive and Computational Neuroscience, Center of Biomedical Technology, Polytechnical University – Complutense University, Madrid, Spain; ^3^Centre for the Prevention of Cognitive Impairment, Madrid, Spain; ^4^Biological and Health Psychology Department, Universidad Autónoma de Madrid, Madrid, Spain

**Keywords:** subjective cognitive decline, older adults, cognitive training, sentence comprehension, naming, executive functions, working memory, cognitive reserve

## Abstract

The present study explores if cognitive reserve, executive functions, and working memory capacity are predictive of performance in the language domain (specifically in sentence comprehension and naming) after a cognitive training intervention. Sixty-six Spanish older adults voluntarily participated in the study, classified either as older adults with subjective cognitive decline according to Jessen et al.’s (2014) criteria (*n* = 35; 70.94 ± 4.16 years old) or cognitively intact (*n* = 31; 71.34 ± 4.96 years old). Written sentence comprehension and visual confrontation naming were assessed both immediately after recruitment (at the baseline), and then 6 months later, once each participant had completed his/her cognitive training (a well-known program in Spain, called UMAM; English translation: Madrid City Council Memory Unit Program). Cognitive reserve, executive functions (cognitive flexibility and controlled interference efficiency), and working memory capacity were measured for all participants at the baseline. Results pointed out that the subjective cognitive decline group presented greater benefits in the language domain than cognitively intact participants. We also observed that lower executive functioning and working memory capacity at the baseline predicted larger benefits in language performance after training, but only in the group of cognitively intact older adults. However, selected predictors hardly explained subjective cognitive decline participants’ results in language performance after training.

## Introduction

Nowadays there is an increasing interest in non-pharmacological intervention effects on older adult cognition. Some studies have shown that cognitive training (CT) is beneficial for older adults’ memory (Gross et al., 2012; Rosi et al., 2017) but also for other domains in this population, as for attention (Rahe et al., 2015), working memory, reasoning and language comprehension (Carretti et al., 2013; Karbach and Verhaeghen, 2014), processing speed and reasoning (Willis and Caskie, 2013), or executive functions (Jackson et al., 2012; Mowszowski et al., 2016); CT is also beneficial for older adults with subjective cognitive decline (SCD) or people with mild cognitive impairment (Martin et al., 2011; Rebok et al., 2014; Smart et al., 2017).

Another issue that requires attention is the one concerning the factors that are strongly associated with greater benefits after CT application. For example, lifestyle factors, occupation attainment or education (cognitive reserve proxies) influence individuals’ cognition along life (Stern, 2012). In this vein, Mondini et al. (2016) have shown that cognitive reserve (CR) can modulate the general cognitive status (measured by Mini-Mental State Examination; MMSE onward) after a CT program and should be considered as a predictor of CT efficacy in older adults with mild to moderate dementia. In Spain a recently published study (Lojo-Seoane et al., 2018) have shown the influence of CR on episodic memory, WM, and general cognitive performance in adults with subjective cognitive complaints evaluated at baseline and at a follow-up after an interval of about 18 months. The results exhibited the positive effect of CR on cognitive performance at baseline and at follow-up, confirming the mediating role of WM on episodic memory and general cognitive performance. Another study (Clark et al., 2016) has indicated that educational attainment modulates the effectivity of training in cognitively intact (CI) older adults, being participants with low educational level the ones with the best performance after training. These and other examples reveal that there are important factors at the baseline which might modulate CT gains, both general or/and in specific cognitive domains (Kraemer et al., 2002; Ranganath et al., 2011). In a study conducted by Bamidis et al. (2015) with a community-dwelling sample of CI and cognitively impaired older adults, the authors reported a robust modulation (inverse) effect of executive functions’ baseline performance on training benefits in global cognition. Additionally, López-Higes et al. (2018) have shown a compensation effect regarding general cognitive status in SCD participants with lower interference efficiency at the baseline. Following the administration of a task-switching intervention in three different populations (children, young, and older adults), Karbach et al. (2017) demonstrated that this training program led to a reduction of age differences. They also observed that baseline abilities predicted both training and transfer gains. Specifically their results showed a compensation effect, since those participants with lower abilities were the ones who improved the most. However, there are other studies that have found opposite results. For example, Guye et al. (2017) have demonstrated very limited evidence for individual differences as predictors of training outcomes, although they found that cognitive performance at baseline was related to training improvement. In particular, those with higher abilities seemed to benefit more, indicating a magnification effect, which was especially apparent in their young adult population.

An additional related issue that has generated a considerable debate is the question of whether and how CT may produce improvements in untrained tasks (see for example, Karbach and Verhaeghen, 2014; Au et al., 2015, 2016; Greenwood and Parasuraman, 2016, for evidence in favor to transfer effects; see also, Melby-Lervåg and Hulme, 2013 or Simons et al., 2016, for results against the existence of such effects). Carretti et al. (2013) have investigated the efficacy of a verbal WM training program in CI older adults, considering the specific training-related benefits in a verbal WM task (criterion) and the transfer effects on measures of WM updating, reasoning, and on language comprehension. They found that the trained older adults performed better than controls in the criterion task and retained this benefit 6 months later. Transfer effects were seen in reasoning and language comprehension performance and were substantially maintained at the 6-month follow-up. In the same line, a study conducted by Payne and Stine-Morrow (2017) examined the effects of a novel home-based computerized CT program focused in verbal WM in healthy older adults in comparison with an active component-control group. Participants in the WM training group showed non-linear improvements in performance on trained verbal WM tasks. In contrast with the control group, WM training participants also showed improvements on untrained verbal WM tasks and across untrained dimensions of language, including sentence memory, verbal fluency, and comprehension of syntactically ambiguous sentences.

In Spain, a well-known multifactorial CT program, called *UMAM* by its name in Spanish [(programa de la Unidad de Memoria del Ayuntamiento de Madrid); English translation: Madrid City Council Memory Unit Program; Montejo et al., 2013] has proven its efficacy on subjective and objective memory measures (and also in mood) in older adults without cognitive impairment, both in the post-training assessment and at 6-month follow-up (Montejo, 2015). The benefits of the UMAM CT program on other cognitive domains, such as language, have been explored in a previous study comparing *APOE* ε4 carriers and non-carriers (López-Higes et al., 2017). That study revealed that there were consistent treatment benefits in complex sentence comprehension, but only in the *APOE* ε4 non-carrier group.

The present study explores the role of CR and executive functions at the baseline as factors which might modulate the efficacy of the UMAM program on complex sentence comprehension and naming in CI and in SCD older adults. The three main executive functions that have been used as predictors in this study: (a) alternation (cognitive flexibility), (b) updating and monitoring of WM representations, and (c) inhibition (as they appear in Miyake et al., 2000 model). In the Miyake and Friedman (2012) model, the part equivalent to the inhibition of the previous model would be a common part of all the executive functions, while the other two, updating and monitoring as well as alternation, would maintain a corresponding specific part within the model. Comprehension and naming are abilities related to successful daily living, since they are needed in various everyday activities and are relevant to older adults’ health and well-being. If older adults with SCD have higher risk of developing dementia (Jessen et al., 2014), it is of great interest to investigate benefits of CT in this population in comparison with CI participants, as well as to find which factors might be associated with greater gains subsequently. Considering a recent work where results evidenced that the UMAM CT program was most beneficial for SCD participants in explaining that their efficiency in inhibition support their general cognitive performance, while the Cis’ WM capacity was the most important performance predictor (López-Higes et al., 2018), our hypothesis are as follows: (1) We expected that CR will not have a significant role as predictor of CT outcomes in language in any group; (2) We hypothesized that sentence comprehension and naming will be most improved in the SCD’s participants after the CT intervention than in those pertaining to the CI’s older group; (3) Provided that the UMAM program is multifactorial, we anticipate a positive effect on participants’ executive functioning which, in turn, will explain linguistic performance; Finally, we assume that program effects will be different depending on the group considered. In short, within the group of older adults with SCD a greater weight of inhibition is expected as a predictor/mediator of linguistic performance, while in the group of CI participants the variable with the greater weight will be a measure related to updating and monitoring representations in WM.

## Materials and Methods

### Participants

Sixty-six older Spanish-speaking adults voluntarily participated in the study. They were recruited from the Center for the Prevention of Cognitive Impairment (a public health institute in Madrid) and, were also enrolled in the UMAM program.

General inclusion criteria to participate in the study were: (1) Normal performance in the Logical Memory delayed recall subtest of the Wechsler Memory Scale – Third Edition (Wechsler, 1997; more than 10 units for people with 16 years of formal education or more; and more than six units for people with 8–15 years of formal education); (2) Yesavage Geriatric Depression Scale (GDS-15; Sheikh and Yesavage, 1986) lower or equal to 9; (3) MMSE (Folstein et al., 1975; Spanish adaptation by Lobo et al., 1999) higher than 24 points at the baseline. All participants had normal or corrected hearing and vision.

**FIGURE 1:**
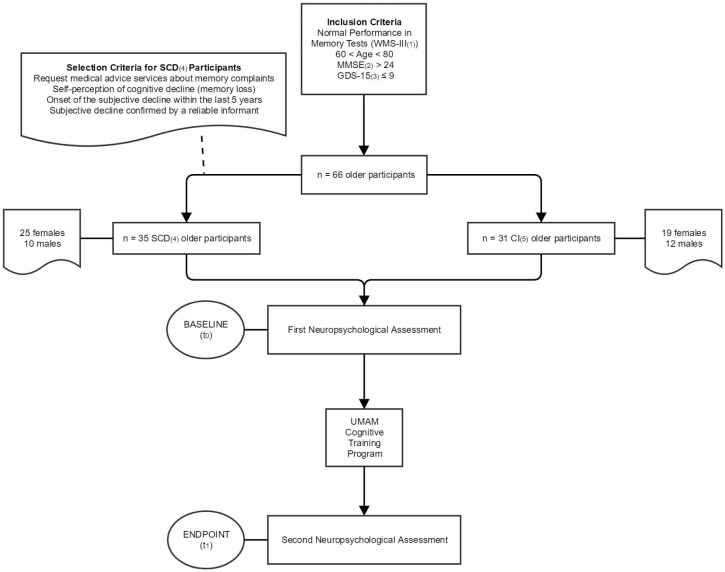
Flowchart showing the inclusion/selection criteria of the 66 participants. All of them were given two neuropsychological assessments: at baseline (0 months) and at the endpoint (6 months). The UMAM cognitive training program was applied between the baseline and the endpoint period. (1) Wechsler Memory Scale III. (2) Mini-Mental State Examination. (3) Geriatric Depression Scale-Short Version. (4) Subjective Cognitive Decline. (5) Cognitively Intact.

Thirty-five participants were identified as older adults with SCD (10 males and 25 females) according to Jessen et al.’s (2014) criteria, that is: (a) they had requested medical advice services about their memory complaints; (b) they presented self-perception of cognitive decline, mainly associated with memory loss; (c) they felt that their subjective decline affected daily life activities; (d) they set the onset of their subjective decline within the last 5 years, and (e) concerns associated with their subjective decline were confirmed by a reliable informant. The remaining 31 participants (12 males and 19 females) did not meet criteria for SCD, and they formed a group of CI older adults. The selection process described along these last lines is summarized in **Figure 1**.

**Table 1** shows the descriptive statistics for socio-demographic variables (age, years of formal education) as well as scores of global cognitive status (MMSE), depressive symptomatology (GDS-15), CR, executive functions and WM capacity in both groups at the baseline. SCD older adults had significant lower scores in interference efficacy as a group than CI participants [*F*(1, 64) = 5.948; *p* = 0.018; $\eta_{p}^{2}$ = 0.016; observed power = 0.669]. No significant differences between groups arose for any other variable.

**Table 1:** Descriptive statistics for socio-demographic variables (age, years of formal education) as well as scores of, global cognitive status (MMSE), depressive symptomatology (GDS-15), cognitive reserve, executive functions and working memory capacity in both groups at baseline.

	CI		SCD	
	Mean	SD	Mean	SD
Age	70.94	4.16	71.39	4.96
Years of formal education	14.38	5.88	13.02	6.05
MMSE	28.94	1.19	28.45	1.50
GDS-15	1.53	2.20	2.55	2.39
Cognitive reserve	15.40	3.85	13.59	4.37
Ratio TMT	2.29	1.08	2.39	0.93
Interference	9.07	7.17	4.71	6.66
Digit reordering	12.68	2.07	11.95	2.25

All participants were informed about the objectives of the study and were invited to participate after signing a written informed consent form. The present study complied with the ethical standards of the Declaration of Helsinki and was approved by the Ethical Committee of the San Carlos Clinical Hospital in Madrid, which is one of the main medical institutions that participates in the current research project.

### The UMAM Cognitive Training Program

The UMAM program was initially designed and implemented in 1995 by the Memory Training Unit of the City Council of Madrid (Unidad de Memoria del Ayuntamiento de Madrid: UMAM). More than 20,000 older adults have benefited from this CT program. This multifactorial intervention consists of 30 sessions that has a duration of 90 min each. Twenty-eight sessions are carried out along 3 months followed by two additional maintaining-booster sessions. Training is organized in groups of 12–18 participants that come to the center in three mornings per week. A typical session begins with 10 min of relaxation, 15 min for homework and other activation activities, followed by 60 min of CT of specific activities, and concluded with suggested homework for the next day (5 min).

The UMAM program is organized in modules:

1. *Module of cognitive stimulation and learning of specific strategies*. This module stimulates basic cognitive processes such as attention, concentration, or perception. The most important memory strategies are taught and practiced: visualization, association, categorization, elaboration, etc. Basic cognitive processes are stimulated by pursuing specific objectives and working on with specific exercises involving attention (focused attention, attentive listening) or perception (a basic process in the registration phase of memorization). Exercises include visual and auditory material. Language acts as mediator in the processes related to codification and retrieving information from memory. Through language exercises the program aims to increase verbal fluency as well as encourage evocation.

The main strategies that are worked on in the program are visualization, association, categorization, and repetition.

2. *Module of memory concepts*. In this module professionals present the most important aspects of memory functioning, highlighting those whose knowledge may have an impact on daily life: types and phases of memory, memory in the elderly and factors that affect memory performance.

3. *Application module to daily life*. It is about practicing and adapting memory strategies to the most frequent daily forgetfulness. In addition, specific techniques are taught and practiced for some memory problems: forgetting where something has been put, forgetting names, understanding and remembering texts, etc. The UMAM program includes techniques that are of interest for the daily forgetfulness in this population. The method of the 3 “R” is emphasized, since it is a procedure to improve the comprehension and memory of texts, as well as the use of an specific technique to recall names. Another aspect that we also deal with is the use of external aids, resources such as diaries, alarms or warnings, telephone directory, putting notes, lists, labels, etc.

From the first session, it is highlighted the importance of reflecting on one’s own daily memory failures (causes and related variables; metamemory) and on the methods used to avoid or overcome them. The UMAM program has been analyzed during its years of implementation, always obtaining good results. The most recent evaluations of this program, such as that observed by of Montejo (2003) where 1,083 users over 65 years of age show that the UMAM program produced a 40% improvement in objective memory in 77% of the cases, and on a subjective level they improved until the 75%, a percentage that was even maintained along the 6 months follow up. In another sample of 2,553 users (Montejo et al., 2013) the normal pre-treatment memory level was 24.8%, reaching a post level of 56%. As mentioned before, the program has proven its efficacy in older adults without cognitive impairment, both in the post-training assessment and at 6-month follow-up (Montejo, 2015).

### Materials

#### Predictor Variables (CR and Executive Functions)

Cognitive reserve was estimated using the CR Questionnaire (CRQ; *Cuestionario de Reserva Cognitiva;* Rami et al., 2011), a brief questionnaire well suited for clinical context. Inhibition efficiency and alternation (cognitive flexibility) were assessed by the Stroop test (Golden, 1978) and the Trail Making Test parts A and B (TMT-A and TMT-B; Reitan, 1994), respectively. The assessment protocol also included a digit reordering task (MacDonald et al., 2001), which involves maintaining and manipulating information in WM (Hill et al., 2010).

#### Dependent Variables (Naming and Sentence Comprehension)

The Boston Naming Test (Kaplan et al., 1983) was selected to explore word retrieval by visual confrontation naming. Sentence comprehension was assessed by means of a sentence–picture simple verification task included in the ECCO_Senior test (*Exploración Cognitiva de la Comprensión de Oraciones para mayores*; English translation: Cognitive Assessment of Sentence Comprehension for seniors; López-Higes et al., 2012). This test includes 36 semantically reversible sentences that are either congruent or incongruent with the picture that accompanies them. Spanish is a Subject–Verb–Object (SVO) language (e.g., *El hombre persigue al perro*; English translation: *The man chases the dog*). Non-canonical word order (as for example in passive sentences: *The man is kicked by the woman*) or number of propositions in a sentence (one in *The girl kissed her boyfriend* vs. two in *The woman kissed by her grandmother was sitting in a chair*) are factors that makes sentences’ processing and interpretation more difficult (Thompson and Shapiro, 2007).

### Procedure

An extensive neuropsychological assessment of each participant, including different cognitive domains (memory, executive functions, language), was conducted at two different times, one immediately after recruitment (*baseline*) and then 6 months later (post-training measure or *endpoint*). Neuropsychological assessments were conducted by an experienced psychologist or psychiatrist at the Center for the Cognitive Impairment Prevention. In the first session, the participants completed the screening tests (MMSE, GDS-15) and CRQ. In addition they were informed about the main goals of the study and signed an informed consent document. All the remaining neuropsychological and cognitive tests were applied in two additional sessions of 50 min each (approximately). Although there was a fixed block of tests arranged for each session, the order in which the tests were presented was randomized in each session for each participant. Tests were applied and scored following the instructions provided in the users’ manuals.

### Statistical Analysis

First, we computed descriptive statistics by group across dependent variables in the baseline and in post-training phases and across five categories of outcomes (percentages). We conducted a repeated measures ANOVA to explore intragroup differences between the baseline and the endpoint across dependent variables. Differences between groups at the baseline across dependent measures or across other relevant factors (age, CR, etc.), as well as those taking into account a measure of training effects (differences between post- and pre-measure in each dependent variable), were computed by means of General Linear Model ANOVAs. Effect sizes were estimated by means of partial eta-square ($\eta_{p}^{2}$); observed power was also given when is needed. In all the analyses we used IBM SPSS statistical program v. 22.0. Regarding predictor variables, we obtained a total score for each participant reflecting an estimation of her/his CR. With respect to digit reordering, we considered the number of series correctly ordered by each participant as a measure of his/her WM capacity (reflecting updating and monitoring of WM representations). With respect to TMT, we used the B/A ratio score (proposed by Lamberty et al., 1994) since it tries to eliminate the effect of the visual search and the perceptomotor speed also present in part B, to measure only the effect of the executive function, finding significant correlations with other tasks that measure alternation or task-switching ability (Arbuthnott and Frank, 2000). Other authors (Maruta et al., 2011) have considered that part B is a common and sensitive measurement of cognitive flexibility (see also Lezak, 1995). That is why we have assumed that the ratio B/A is a more fine-grained score of that function. Finally, with regards to the Stroop test, we used the Interference index proposed by Chafetz and Matthews (2004). In order to test the role of different predictors regarding CT outcomes in the language domain we have followed a procedure described in Mondini et al. (2016). The measures employed in the analyses resulted from subtracting the baseline scores to the endpoint scores in each of the selected dependent variables. All resulting models were compared using four indexes providing goodness-of-fit measures: Bayesian Information Criterion (BIC), Akaike Information Criterion (AIC), Bayes Factor (BF), and *R*^2^. Higher BF and *R*^2^ values and lower BIC and AIC indexes, denote better model fit to data. BIC, AIC and *R*^2^ were computed using IBM SPSS statistical software v. 20.0, but the Bayes Factor index was computed with version 0.9.8 of the BayesFactor package developed by Perception and Cognition Lab, Department of Psychological Sciences at the University of Missouri^1^.

## Results

### Baseline Comparisons Between Groups

**Table 2** shows means and standard deviations (*SD*) by group across language performance measures (pre and post).

**Table 2:** Mean and standard deviation (in brackets) in dependent measures at the baseline (pre-) and at the endpoint (post-training) for both groups.

	Baseline (pre)			Endpoint (post)		
	Non-canonical sentences	Sentences with two propositions	BNT	Non-canonical sentences	Sentences with two propositions	BNT
CI	14.91 (1.91)	15.41 (2.30)	54.26 (3.79)	15.26 (1.93)	16.32 (1.77)	55.06 (3.65)
SCD	13.79 (2.29)	14.72 (2.63)	50.39 (6.31)	14.31 (1.86)	14.74 (2.21)	52.15 (4.86)

At the baseline the CI group presented a significant better performance in naming [*F*(1, 64) = 8.174; *p* = 0.006; $\eta_{p}^{2}$ = 0.106; observed power = 0.805] than the SCD group. Differences in sentences not fitted to canonical word order in Spanish almost reached statistical significance [*F*(1, 64) = 3.724; *p* = 0.058; $\eta_{p}^{2}$ = 0.051; observed power = 0.477].

### Training Outcomes Differences

Intragroup analysis in the CI group showed that the difference between pre- and post-training measures in sentences including two propositions reached statistical significance [*F*(1, 30) = 5.025; *p* = 0.033; $\eta_{p}^{2}$ = 0.145; observed power = 0.583]. However, in the SCD group the analysis revealed that performance in naming improved after cognitive training [*F*(1, 34) = 12.612; *p* = 0.001; $\eta_{p}^{2}$ = 0.240; observed power = 0.934]. Effect sizes in both cases were large, but especially in SCD older adults.

No significant differences between groups were observed in any of the scores representing the effect of training (post – pre) in the three dependent measures.

### Training Outcomes Categorization

Once differences in measures between post and pre-training were computed for all participants, it was possible to recode values in five categories of outcomes: (a) a positive value greater or equal to one SD was considered as moderate improvement, (b) positive values between 0 and 1 SD were mild improvement, (c) a value equal to zero meant null improvement, (d) negative values between 0 and 1 SD formed a category of mild worsening, and (e) negative values greater or equal to 1 SD were considered moderate worsening. **Table 3** summarizes percentages in these five categories by group. Pearson Chi-square did not reach statistical significance in any case [χ^2^(4) = 0.664, *p* = 0.324; χ^2^(4) = 1.500, *p* = 0.827; χ^2^(4) = 4.857, *p* = 0.302].

**Table 3:** Percentages of cases across outcome categories in CI and in SCD groups.

		CI	SCD
Non-canonical sentences	Null improvement	9.7%	20.0%
	Mild worsening	16.1%	14.3%
	Moderate worsening	19.4%	11.4%
	Mild improvement	35.5%	20.0%
	Moderate Improvement	19.4%	34.3%
Sentences with two propositions	Null improvement	25.8%	31.4%
	Mild worsening	22.6%	28.6%
	Moderate worsening	3.2%	5.7%
	Mild improvement	25.8%	20.0%
	Moderate improvement	22.6%	14.3%
BNT	Null improvement	29.0%	13.2%
	Mild worsening	25.8%	18.4%
	Moderate worsening	0.0%	2.6%
	Mild improvement	32.3%	42.1%
	Moderate improvement	12.9%	23.7%

### Prediction Models and Benefits in Linguistic Performance After CT

Regarding the main objective of the study, **Table 4** shows that for CI participant’s performance on **sentences not fitted to the canonical word order in Spanish** the model with best goodness of fit was the 2nd (BIC = 124.01; AIC = 119.81; BF = 2.20; *R*^2^ = 0.15; *p* < 0.026), with TMT ratio as the only significant predictor (*t* = 2.26, *p* = 0.032). In SCD older adults the best model was the 7th, which includes CR and digit reordering (BIC = 127.87; AIC = 122.68; BF = 1.92; *R*^2^ = 0.11; *p* < 0.030). However, nor CR (*t* = -1.79, *p* = 0.086) neither digit reordering (*t* = 0.55, *p* = 0.583) reached statistical significance.

**Table 4:** Linear regression models for sentences not fitted to canonical word order in Spanish included in ECCO_Senior.

		CI group					SCD group					
Model		*BIC*	*AIC*	*BF*	*R^2^*	*p*	*BIC*	*AIC*	*BF*	*R^2^*	*p*	
0	Intercept	125.65	122.85				128.34	125.75			
1	CR	129.00	124.79	2.84	0.00	0.813	127.29	123.40	1.06	0.10	0.037
2	**TMT**	**124.01**	**119.81**	**2.20**	**0.15**	**0.025**	131.42	127.53	3.04	0.00	0.642
3	Inter	128.89	124.69	2.68	0.01	0.692	129.14	125.26	1.22	0.06	0.115
4	DR	127.80	123.59	2.06	0.03	0.262	130.59	126.70	3.02	0.00	0.307
5	CR + TMT	127.32	121.71	1.10	0.16	0.077	130.37	125.19	2.17	0.10	0.102
6	CR + Inter	132.15	126.55	5.52	0.01	0.861	129.80	124.62	1.07	0.17	0.077
7	**CR + DR**	131.19	125.59	4.04	0.04	0.533	**127.87**	**122.68**	**1.92**	**0.11**	**0.029**
8	TMT + Inter	126.55	120.94	1.19	0.18	0.052	132.27	127.09	1.82	0.10	0.265
9	TMT + DR	126.79	121.18	1.09	0.17	0.059	133.60	128.42	6.19	0.01	0.516
10	Inter + DR	130.84	125.24	1.20	0.05	0.447	130.72	125.54	2.84	0.07	0.122
11	CR + TMT + Inter	129.53	122.52	1.56	0.19	0.097	132.91	126.43	2.05	0.18	0.150
12	CR + TMT + DR	130.16	123.15	1.90	0.17	0.128	130.83	124.35	3.79	0.11	0.060
13	TMT + Inter + DR	129.08	122.07	1.35	0.20	0.079	133.78	127.30	3.77	0.10	0.218
14	CR + TMT + Inter + DR	132.21	123.80	2.41	0.21	0.134	133.10	125.33	1.53	0.27	0.077

*CR, cognitive reserve; TMT, Trail Making Test; Inter, Stroop’s interference; DR, digit reordering; BIC, Bayesian Information Criterion; AIC, Akaike Information Criterion; BF, Bayes Factor. Bolded values correspond to models whit better fit to data*.

**FIGURE 2:**
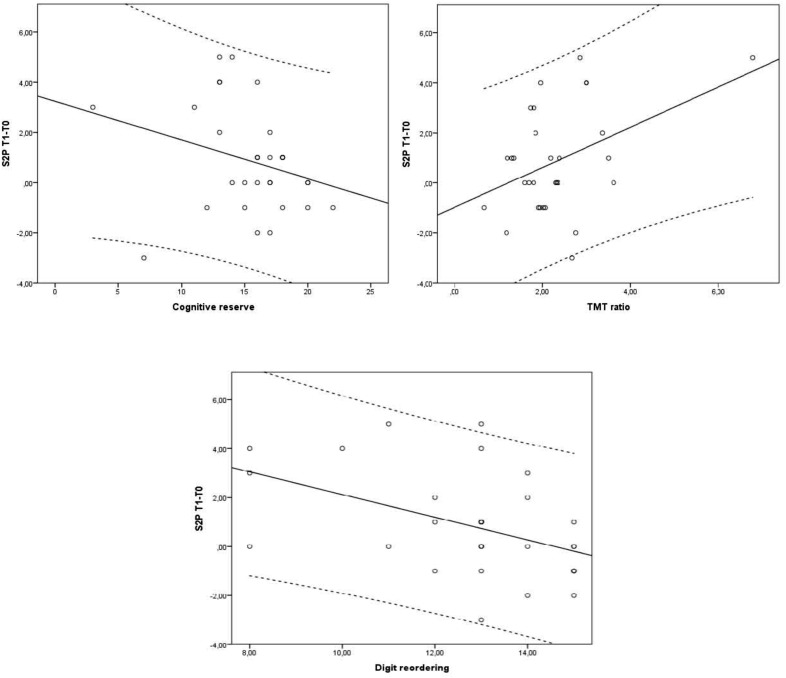
Cognitive reserve, Trail Making Test ratio (TMT ratio) and digit reordering effects on the difference in sentences with two propositions between the endpoint (Tl) and the baseline (TO) for the control group, according to model 12 (**Table 5**). Dotted lines delimit 95% confidence interval.

Considering **sentences with two propositions**, the model with the best goodness-of-fit for the CI group was the 9th (**Table 5**) with TMT ratio and digit reordering as predictors (BIC = 130.15; AIC = 124.55; BF = 12.51; *R*^2^ = 0.34; *p* < 0.003). Both variables made a significant contribution to the model, but with different signs, thus in different directions (TMT ratio: *t* = 2.16, *p* = 0.039; digit reordering: *t* = -2.58, *p* = 0.016). Model 12th also had a good fit, and included as predictors CR, TMT ratio and digit reordering (BIC = 131.29; AIC = 124.28; BF = 10.62; *R*^2^ = 0.39; *p* < 0.003). CR did not contribute in a significant manner to the model (*t* = -1.43, *p* = 0.165), but TMT ratio and digit reordering did it in the same direction than in the previous model (TMT ratio: *t* = 2.28, *p* = 0.031; digit reordering: *t* = -2.32, *p* = 0.028; see **Figure 2**). In the SCD group the 1st model, with CR as the only predictor (see again **Table 6**), only approached significance (BIC = 126.31; AIC = 122.42; BF = 1.13; *R*^2^ = 0.08; *p* < 0.052). CR did not have a significant role (*t* = -1.58,*p* = 0.126).

**Table 5:** Linear regression models for sentences with two propositions included in ECCO_Senior.

		CI group					SCD group					
Model		*BIC*	*AIC*	*BF*	*R^2^*	*p*	*BIC*	*AIC*	*BF*	*R^2^*	*p*	
0	Intercept	135.80	133.00				126.83	124.24			
1	**CR**	136.73	132.53	1.18	0.08	0.116	**126.31**	**122.42**	**1.13**	**0.08**	**0.051**
2	TMT	133.37	129.17	2.98	0.18	0.016	130.09	126.20	2.79	0.01	0.833
3	Inter	139.14	134.94	2.90	0.00	0.804	127.71	123.82	2.40	0.02	0.120
4	DR	131.55	127.35	5.02	0.21	0.006	130.11	126.22	2.60	0.01	0.876
5	CR + TMT	133.53	127.92	3.63	0.26	0.011	129.57	124.39	2.54	0.09	0.145
6	CR + Inter	140.07	134.47	2.64	0.08	0.282	128.78	123.59	1.26	0.16	0.098
7	CR + DR	133.37	127.77	3.85	0.26	0.010	129.21	124.03	2.46	0.09	0.122
8	TMT + Inter	136.71	131.11	1.17	0.18	0.053	130.98	125.80	5.11	0.02	0.295
9	**TMT + DR**	**130.15**	**124.55**	**12.51**	**0.34**	**0.002**	133.35	128.17	5.27	0.02	0.964
10	Inter + DR	134.92	129.31	1.91	0.21	0.021	130.86	125.67	5.43	0.02	0.277
11	CR + TMT + Inter	136.12	129.11	2.05	0.28	0.020	132.05	125.57	2.50	0.16	0.198
12	**CR + TMT + DR**	**131.29**	**124.28**	**10.62**	**0.39**	**0.002**	132.46	125.98	4.71	0.09	0.234
13	TMT + Inter + DR	133.16	126.15	5.51	0.35	0.005	134.13	127.65	9.83	0.02	0.459
14	CR + TMT + Inter + DR	133.29	124.88	7.10	0.42	0.003	134.86	127.09	3.92	0.17	0.272

*CR, cognitive reserve; TMT, Trail Making Test; Inter, Stroop’s interference; DR, digit reordering; BIC, Bayesian Information Criterion; AIC, Akaike Information Criterion; BF, Bayes Factor. Bolded values correspond to models whit better fit to data*.

**Table 6:** Linear regression models for the Boston Naming test.

		CI group					SCD group					
Model		*BIC*	*AIC*	*BF*	*R^2^*	*p*	*BIC*	*AIC*	*BF*	*R^2^*	*p*	
0	Intercept	144.30	141.50				160.20	157.46				
1	CR	147.69	143.48	2.90	0.00	0.907	160.10	155.99	1.28	0.07	0.063	
2	TMT	147.65	143.45	2.87	0.00	0.821	162.09	157.98	1.23	0.06	0.224	
3	**Inter**	**143.11**	**138.90**	**2.75**	**0.17**	**0.032**	163.55	159.45	3.08	0.00	0.916	
4	DR	146.45	142.24	1.55	0.05	0.263	163.14	159.04	2.09	0.03	0.514	
5	CR + TMT	151.04	145.43	5.97	0.00	0.969	161.30	155.83	1.01	0.16	0.060	
6	CR + Inter	146.16	140.56	1.17	0.15	0.085	162.97	157.50	1.61	0.13	0.138	
7	CR + DR	149.84	144.24	4.04	0.04	0.532	162.36	156.89	2.79	0.07	0.102	
8	TMT + Inter	146.00	140.39	1.28	0.18	0.078	165.40	159.93	3.01	0.06	0.464	
9	TMT + DR	149.65	144.04	3.19	0.06	0.483	165.14	159.67	1.45	0.11	0.408	
10	Inter + DR	145.77	140.17	1.39	0.19	0.070	166.47	161.01	5.42	0.02	0.795
11	CR + TMT + Inter	148.96	141.96	1.99	0.17	0.136	164.29	157.45	1.71	0.19	0.111
12	CR + TMT + DR	153.03	146.03	7.08	0.05	0.689	163.71	156.88	1.97	0.17	0.086
13	TMT + Inter + DR	148.42	141.41	1.20	0.21	0.107	168.42	161.58	4.30	0.09	0.597
14	CR + TMT + Inter + DR	151.11	142.70	2.58	0.20	0.147	166.74	158.53	2.43	0.21	0.140

*CR, cognitive reserve; TMT, Trail Making Test; Inter, Stroop’s interference; DR, digit reordering; BIC, Bayesian Information Criterion; AIC, Akaike Information Criterion; BF, Bayes Factor. Bolded values correspond to models whit better fit to data*.

Finally, when **visual confrontation naming** was considered (**Table 6**), the best model for the CI group was the 3rd (BIC = 143.11; AIC = 138.90; BF = 2.75; *R*^2^ = 0.17; *p* < 0.033), with interference as the only significant predictor (*t* = -2.40, *p* = 0.023). None of the regression models computed for this dependent measure in the SCD group reached statistical significance.

### How Single Predictors Explain Language performance After CT

When sentences not fitted to canonical word order in Spanish are taken into account (see **Table 4** again) there was a clear difference between groups, since in CI participants TMT ratio explains a 15% of criterion’s total variance, whereas CR was the variable which explains more than the other predictors (10%) in the SCD group. Regarding sentences with two propositions, digit reordering was the predictor with the highest explanation power in the CI group (it explains by itself 21% of the total variance), whereas CR (once again) explains only 8% (**Table 5**) in SCD participants. As **Table 6** shows, Stroop interference was the predictor that best explained naming performance after CT in CI participants (17%), and consistently with previous results, CR explained 7% of criterion in the SCD group.

## Discussion

The present study aims to draw some conclusions regarding UMAM benefits in the language area. Based on our results, we might say in first place that the UMAM program produces effects in abilities related to older people’s daily life, that is, in sentence reading comprehension and naming. Considering the percentages of moderate improvement (of greater relevance from a clinical point of view), as well as the resulting differences between groups in CT outcomes and intra-group differences between pre- and post-training measures, we can conclude that SCD is the group in which benefits are greater, confirming one of our previous hypothesis. This group considerably improves their results in naming (in which we have observed the largest effect size) and, in to a lesser extent, in sentences not fitted to canonical word order in Spanish at the post-treatment phase. It should be noted that these results are especially favorable for UMAM program, since the older adults with SCD had at baseline greater susceptibility to distraction or lesser efficiency in inhibiting irrelevant information (assessed with the Stroop test) than the CI group, as well as worse results in the two aforementioned indicators. In the post-training phase, the two groups showed an equivalent performance. Some studies have reported similar benefits in language comprehension, but after a verbal WM training program which was applied to CI older adults aged 65–75 years (Carretti et al., 2013). Those results are also in line with that obtained in the study conducted by Payne and Stine-Morrow (2017) using a novel home-based computerized CT program focused on verbal WM, and comparing CI older adults with a control group. The UMAM program includes vocabulary exercises, and other requiring reading texts or following instructions, which are all related to written language comprehension. These features can lead to positive training outcomes associated with sentence comprehension skills and naming, and could be considered as near transfer effects.

With respect to the main objective of the study, that is, to explore the role of CR and executive functions as factors which could modulate or predict the efficacy of the UMAM program in language (complex sentence comprehension and naming), there is a clear difference between the two groups. With respect to non-canonical sentence comprehension the results for the CI group emphasize that high TMT ratios (meaning low cognitive flexibility) are significantly related to high CT benefits (since *t* statistical test value was positive), and that this variable explains 15% of the total criterion variance. However, in the SCD group although we found a model including CR and digit reordering which explained 11% of the total variance, none of these variables reached statistical significance. Thus, the results show that CT outcomes regarding sentences not fitted to canonical word order in Spanish were higher for those CI older adults who had lower cognitive flexibility scores at baseline.

Furthermore, CI older adults who had higher TMT ratio scores (low cognitive flexibility) and lower digit reordering scores at baseline, also showed a significant improvement in their performance after CT in sentences with two propositions. These two factors explain 34% of the total variance (reaching 39% if CR is added), so their weight in sentence comprehension is moderately high. This result is consistent with that obtained in a good number of studies that show the important role of working memory in understanding complex sentences, especially linked to the number of propositions in the sentence (Waters and Caplan, 2004). In the SCD group, CR almost reached statistical significance for this type of sentences, presenting a slight tendency implying better training outcomes for participants with lower CR at baseline.

Regarding participants’ performance in naming, the results pointed out that CI older adults with lower inhibition efficiency at baseline had better results in BNT after CT, and that this factor explains 17% of the total variance associated with criterion. However, in the SCD group none of the models computed for this dependent measure reached statistical significance.

These results enable drawing another conclusion: executive functions at baseline (Ranganath et al., 2011) play a role as predictors of language performance after training, but only in CI older adults. The association between executive functions at the baseline and CT benefits is especially important in complex sentence comprehension (exemplars with two verbs or propositions) and, to a lesser extent, in naming, but restricted to older adults in the CI group. Selected predictors hardly explain SCD older adults’ post-training performance, so a substantial part of its variance remained unexplained, suggesting that other variables not considered in the study may account for it. This pattern of results contradicts, at least in part, our hypothesis regarding the role of executive functions in both groups as factors explaining language performance.

Although CT benefited language performance in older adults with SCD more than in CI participants, the chosen factors (executive functions) did not explain these benefits. On the contrary, the same factors predicted the benefits of CT for CI individuals. This is an paradoxical pattern of results that demand an explanation. It has been suggested that CT may cause a decrease in depressive symptomatology, or an improvement in general mood, well-being or motivation in SCD participants’ performance (Bherer, 2015). Therefore, the aforementioned changes, could be responsible for the observed improvement in the area of language in the group of older adults with SCD. Other authors have suggested that a change in lifestyle (variety of cognitive, physical, and social activities) associated to voluntary participation in training program has positive effects in global cognition and memory (Küster et al., 2016). Some studies have also found effects of multitasking training on processes such as perception and attention (Anguera et al., 2013), which are basic for others of higher level, so they could also be considered as factors to be taken into account. A complementary explanation arise when the pattern of differences across predictors (in isolation) are observed: (a) CR has a marginal weight as a factor explaining language performance in the SCD group (also in the CI group); (b) However, in the group of CI older adults two specific executive functions, alternation (cognitive flexibility) and updating and monitoring (evaluated by digit reordering), explain a significant portion of the variance associated to sentence comprehension. Inhibition efficiency, a common part for all the executive functions (following Miyake and Friedman model), explains a substantial portion of naming performance in this later group. Thus, it seems that UMAM program enhances CI older adults’ executive functioning, which benefits, in turn, CI participant’s performance in the linguistic tasks considered here (many studies have pointed out close relationships between executive functions and language, especially in the process of reanalysis of complex sentences; see for example: Caplan and Waters, 2013; Yoon et al., 2015). This would be an adequate explanation given that, as some authors maintain (for example: Lustig et al., 2009), intervention programs that incorporate multiple strategies and a great variety of tasks produce greater (near) transfer effects. It should bear in mind that the UMAM program includes vocabulary exercises, and other requiring reading texts or following instructions, which are all related to written language comprehension. In summary, four main conclusions can be drawn of this study. Firstly, the ECCO_Senior test has been revealed as a screening instrument with enough sensitivity to detect differences in written language comprehension among adults with and without SCD. Secondly, older adults with SCD obtain greater benefits in the language domain after training in comparison to CI older adults. However, near transfer effects to language in the elderly group SCD cannot be explained adequately with the predictors selected in this study. Thirdly, the UMAM program seems to enhance CI older adults’ executive functioning, which benefits, in turn, CI participants’ performance in sentence comprehension and naming. Finally, as we expected, CR has a marginal role as predictor of participants’ linguistic performance.

Since we have obtained results that contrast with other studies which have also considered cognitive functioning at baseline (Guye et al., 2017), future research should address if these differences might emerge as a function of the population considered, the type of training, or other factors such as motivation, etc. A possible line of future research would involve exploring the role of other factors/predictors such as those that have been suggested to explain the pattern of results obtained in the group of older adults with SCD; it would involve to study the changes that occur in mood, general well-being or daily activity (physical, social, intellectual) in this population for their participation in the UMAM program, as well as the relevance of these variables in the prediction of SCD participants’ performance in different cognitive domains, including language. Likewise, it would be interesting to explore the relationships between executive functions and language in cognitively intact older adults, checking if the training program produces a significant improvement in the executive functions (especially those which are more related to language), and if this is also related directly with an improvement in language skills.

The UMAM program has demonstrated its efficacy improving memory in older adults with and without SCD and in mild cognitive impairment. From a practical point of view the present results are useful since they allow us to know which factors explain to a large extent the benefits that the UMAM program has in the language area in cognitively intact older adults. On the other hand, the results found in the group of participants with SCD, which reveal important differences with CI older adults in relation to the mechanisms that underlie the benefits produced by the UMAM program in a specific area such as language, serve as an incentive to deepen the study of other predictors that provide an adequate explanation. Taken as a whole, the results of this study could be indicating the need to adapt the UMAM program in a flexible way to the characteristics of the users as a consequence of its location in the continuum that goes from normality to mild cognitive deterioration, and to the specific functions that are intended to maintain or improve.

Potential limitations of this study would be that the percentage of females were greater than the corresponding of males (66% vs. 34%). Similarly, the sample size could also be extended in future studies to strengthen conclusions.

## Author Contributions

Study design and concept, as well as data analyses and interpretation were done by RL-H, JP, and SR-V. Critical revision of results and discussion were done by IR-R, JdF-L, MM, and PM. Evaluation and data collection were conducted by ML and DP.

## Conflict of Interest Statement

The authors declare that the research was conducted in the absence of any commercial or financial relationships that could be construed as a potential conflict of interest.

## Footnotes

1 http://pcl.missouri.edu/bf-reg
